# The Response of Antarctic Sea Ice Algae to Changes in pH and CO_2_

**DOI:** 10.1371/journal.pone.0086984

**Published:** 2014-01-28

**Authors:** Andrew McMinn, Marius N. Müller, Andrew Martin, Ken G. Ryan

**Affiliations:** 1 Institute for Marine and Antarctic Studies, University of Tasmania, Hobart, Tasmania, Australia; 2 School of Biological Sciences, Victoria University of Wellington, Wellington, New Zealand; University of Tasmania, Australia

## Abstract

Ocean acidification substantially alters ocean carbon chemistry and hence pH but the effects on sea ice formation and the CO_2_ concentration in the enclosed brine channels are unknown. Microbial communities inhabiting sea ice ecosystems currently contribute 10–50% of the annual primary production of polar seas, supporting overwintering zooplankton species, especially Antarctic krill, and seeding spring phytoplankton blooms. Ocean acidification is occurring in all surface waters but the strongest effects will be experienced in polar ecosystems with significant effects on all trophic levels. Brine algae collected from McMurdo Sound (Antarctica) sea ice was incubated *in situ* under various carbonate chemistry conditions. The carbon chemistry was manipulated with acid, bicarbonate and bases to produce a pCO_2_ and pH range from 238 to 6066 µatm and 7.19 to 8.66, respectively. Elevated pCO_2_ positively affected the growth rate of the brine algal community, dominated by the unique ice dinoflagellate, *Polarella glacialis.* Growth rates were significantly reduced when pH dropped below 7.6. However, when the pH was held constant and the pCO_2_ increased, growth rates of the brine algae increased by more than 20% and showed no decline at pCO_2_ values more than five times current ambient levels. We suggest that projected increases in seawater pCO_2_, associated with OA, will not adversely impact brine algal communities.

## Introduction

Ocean acidification (OA) is a direct ocean response to anthropogenic CO_2_ emissions and will continue irrespective of whether the planet warms. The increase in atmospheric CO_2_ levels from the present (∼400 µatm) to levels likely by the end of the century (∼700 µatm, [1]), will lead to an increase in average seawater CO_2_ concentration from around 12.5 µmol kg^−1^ to ∼20 µmol kg^−1^. This will be accompanied by a fall in pH from a global preindustrial average of ∼8.2 to 7.8. Current pH values adjacent to Antarctica are 8.019–8.045 [2]. Some microalgal species will benefit from increased CO_2_ concentrations, which will facilitate faster rates of photosynthesis and growth. Although many species, including most diatoms, possess effective carbon-concentrating mechanisms (CCM), they may still benefit from decreased costs for carbon acquisition [3], [4], [5]. Whether global marine primary production will benefit from projected changes in CO_2_ levels will largely be determined by nutrient supply and grazer impact since most oceans are oligotrophic and limited by nutrients and/or light rather than CO_2_ availability [6], [7], [8], [9].

The direct effect of oceanic pH on microalgal cells is less clear but recent work has demonstrated that it can modify intracellular pH and affect membrane potential, energy partitioning and enzyme activity [10], [11]. Many coastal and estuarine phytoplankton are unaffected by external pH changes in the range 7.8–8.5, probably because this range reflects natural diurnal and seasonal changes in many coastal environments [12], [13], [14]. Natural increases in pH to >10 have been reported from dense phytoplankton blooms before limiting access to CO_2_ causes photosynthesis to be impaired. Furthermore, microalgae living in extreme environments are able to tolerate short term exposure over a very large range in pH (3–11) [15] but in the ocean it is likely to be the resultant changes in nutrient availability and not pH itself, which will have the greatest impacts on growth and survival [6].

The situation in sea ice ecosystems, however, is quite different to that in the open sea. During ice formation, internal gas concentrations are heavily modified by exclusion of gases from the ice crystal structure. This results in a steep decline in pH, changes to gas solubility at high salinity and low temperature often resulting in supersaturation and outgassing [16]. Internal liquid seawater temperatures can drop to below −15°C with salinities fluctuating between 173 in winter and 0 in summer [16]. Photosynthetic activity has been recorded at temperatures below −10°C [17]. However, in spring and summer this trend is usually overridden by the photosynthetic activity of dense microalgal communities that cause the pH to rise as a result of the depletion of the dissolved CO_2._ Values of up to 8.9, for instance, have been reported from platelet ice [18] and up to 9.9 in brine channels within the ice matrix [16].

Sea ice microbial communities are a critical component of polar marine ecosystems, contributing 10–50% of the annual primary production of polar seas [19], supporting overwintering zooplankton species, especially Antarctic krill, and seeding spring phytoplankton blooms. Ocean acidification will be experienced most strongly in these ecosystems and is likely to have significant effects on all trophic levels [20]. These communities are a close association between microalgae, heterotrophic bacteria and heterotrophic protists. Algae and other microorganisms proliferate in sea-ice during spring with bacterial production coupled tightly to algal production [21]. In some communities, such as those at the surface of the ice or those in brine channels, cells grow despite exposure to extremes of temperature and salinity, while those on the underside of the ice remain at close to freezing point. Brine channel and surface communities have only limited access to the underlying water column and consequently their CO_2_ (and nutrient) supply becomes severely restricted. Under these circumstances, restricted CO_2_ supply limits microalgae growth [22]. Thus, unlike planktonic ecosystems where CO_2_ is rarely in short supply, in sea ice brine systems the shortage is often acute and the addition of CO_2_ as a result of OA may partially alleviate this stress. Nitrate also becomes less significant as ammonium levels increase at lower pH with enhanced disassociation to ammonia, which is more readily assimilated [18]. In addition, elevated oxygen levels may negatively influence growth [23] as do processes associated with iron and nitrogen transformation [18]. By comparison, algal communities on the undersurface of the ice usually experience replete nutrient conditions and an unrestricted CO_2_ supply. Sea ice microbial communities are already living at physiochemical extremes and additional changes such as a decrease in pH have the potential to shift environmental conditions beyond physiologically tolerable levels.

At its most fundamental level, ocean acidification is about the effects of changing resource availability (i.e. nutrients and CO_2_) coupled with cellular energy demands and environmental changes in pH. Here we report the *in situ* response of ice algal communities to changing carbonate speciation. This study is the first *in situ* study of the response of sea ice brine algae to manipulated carbon chemistry.

## Materials and Methods

The sea ice incubation experiments were conducted on 16–26 Nov, 2012 at Cape Evans, McMurdo Sound (77° 38′S, 166° 24′E), Antarctica. Brine was collected from 0.5 m deep sack holes, drilled with a 20 cm diameter motorised auger at the beginning of each experiment (Jiffy, USA). The temperature and salinity of the brine was measured prior to chemical manipulation and preparation of each treatment, using a digital thermometer (Digitron, 2098T) and a refractometer (HANNA Instruments Inc. Woonsocket-RI-USA, Model: HI96822). After chemical manipulation (see below), five replicates were decanted into clear 250 ml polycarbonate vials with clear lids leaving no headspace. The vials containing the brine algae were placed in 13 cm diameter holes that had been cored to a depth of 0.5 m with a motorised ice corer (Kovacs, USA). The ice cores initially extracted from each hole was subsequently placed on top of the vials, generating a natural light and temperature environment. Irradiance was measured with a Biosphaerical QSP 200 4π light meter.

The work program and sampling protocol for these experiments were approved under the provisions of the Antarctic Treaty by a permit (issued to Ken Ryan) by Antarctica New Zealand. No protected or endangered species were used in the experiments and no specially protected areas were visited. All experiments were conducted *in situ*, in Antarctica.

### CO_2_ Manipulation

The, brine carbonate chemistry was manipulated by the addition of HCl, NaOH and NaHCO_3_. In the first experiment (5 treatments and 5 replicates), total alkalinity (TA) was manipulated while keeping the DIC concentration constant by the addition of HCl or NaOH resulting in a pH (total scale) and pCO_2_ gradient from 7.19 to 8.66 and from 178 to 5095 µatm, respectively. In the second experiment (4 treatments and 5 replicates), the carbonate system was manipulated by addition of NaHCO_3_ and NaOH keeping the pH constant (8.02±0.05) while applying a pCO_2_ gradient ranging from 505 to 2477 µatm.

Samples for dissolved inorganic carbon (DIC) and TA were taken from the bulk samples at the beginning of each experiment and then from the replicates at the end. Samples were taken for DIC, TA, chl-*a*, Coulter Multisizer-analysis and PAM fluorometry at the beginning and end of each experiment. DIC samples were filtered through a sterile filter (pore size 0.2 µm) into 12.5 ml Labco Exetainers® avoiding air contact, poisoned with HgCl_2_ and sealed air tight. TA samples were filtered through GF/F filters (used for chl-*a* analysis), poisoned with HgCl_2_ and stored in 250 ml polyethylene flasks. Samples for Coulter-Multisizer analysis (12 ml) were fixed with Lugol’s solution and stored in the dark until analysed.

The carbonate system was calculated from temperature, salinity, TA and DIC measurements using the program CO_2_sys (version 1.05 by E Lewis and DWR Wallace) with dissociation constants for carbonic acid [24]. DIC and TA samples were analysed as the mean of triplicate measurements with the infrared detection method using an Apollo SciTech DIC-Analyzer (Model AS-C3) and the potentiometric titration method with modification for high alkalinity [25], [26] respectively. Data were corrected to Certified Reference Materials (CRM) (Scripps Institution of Oceanography, USA). Consecutive measurements of the Dickson standard resulted in an average precision of >99.9% for both TA and DIC analysis.

### Coulter Multisizer Analysis

Samples were analysed as the mean of triplicate measurements with a Beckman Coulter Multisizer IV equipped with a 100 µm aperture covering particle volume range from 4 to 113,097 µm^3^.

### Chlorophyll Analysis and Flourometry

Samples were filtered onto 42 mm diameter Whatman GF/F filters. The filters were then extracted in 10 ml of 90% methanol for 12 hours at 4°C in the dark. Chlorophyll-*a* was measured on a Turner 10AU fluorometer using the acidification method [27].

A WaterPAM (Walz GmbH, Germany) was used for the maximum quantum yield (F_v_/F_m_) measurements. Gain settings were between 18 and 22. All samples were dark adapted at −2°C for one hour prior to measurement. One-way ANOVA was used to compare end-point data for the control and treatment.

### Data Analysis

One-way analysis of variance (ANOVA) and Tukey post hoc tests were used to test for significant differences within/among treatments using SPSS v20 (IBM). If heteroscedastic, the data was transformed prior to analysis.

## Results

The sea ice at Cape Evans, McMurdo Sound, in November-December 2012 was 1.7 m thick with no snow cover. The brine community was strongly dominated by the dinoflagellate, *Polarella glacialis*. Maximum midday irradiances were approximately 100–120 µmol photons m^−2^ s^−1^ in the 50 cm deep holes.

### Reduced pH and Increased CO_2_ Experiment

**Figure 1:**
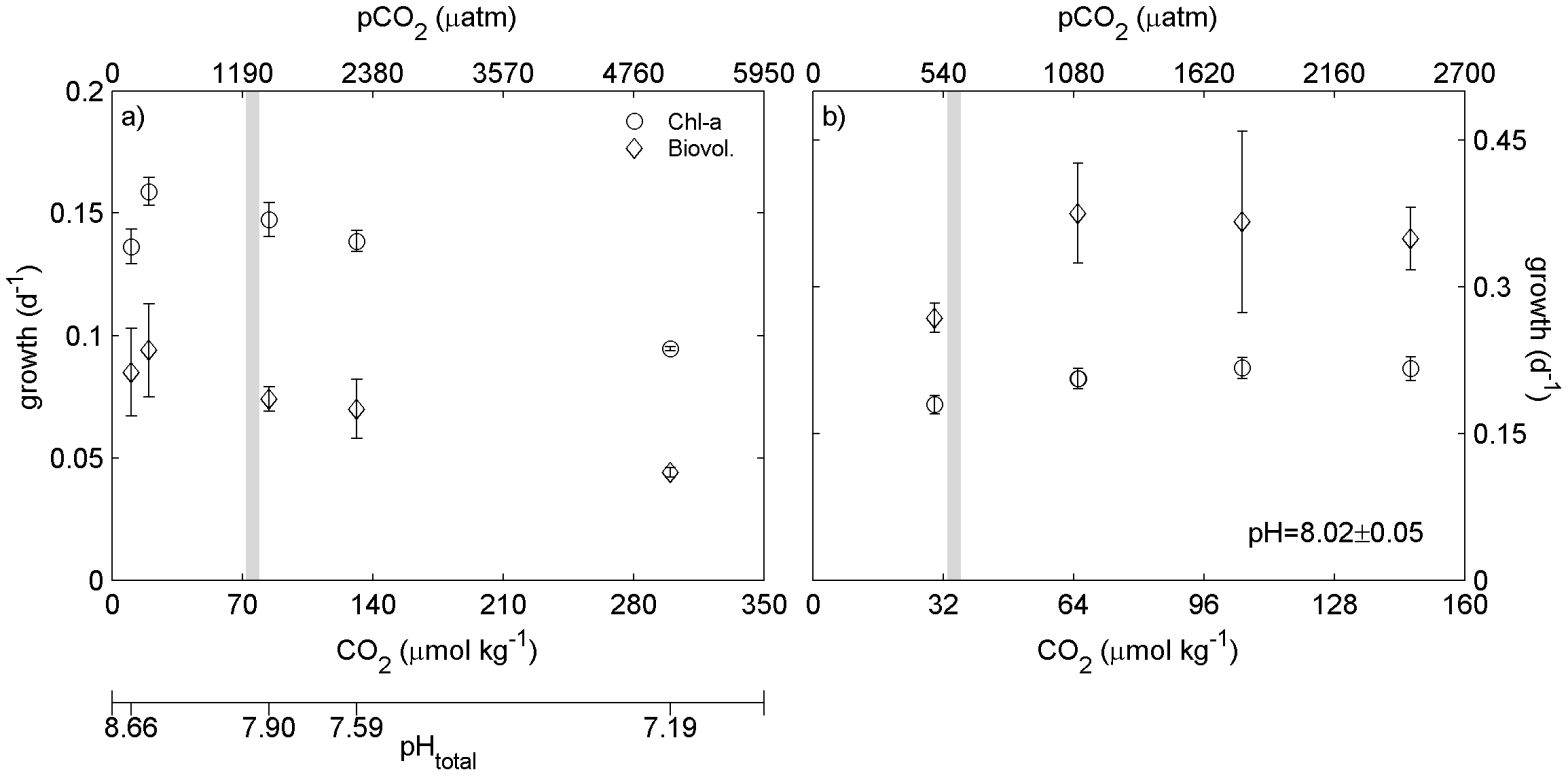
Growth rate changes in sea ice brine algae based on chl-*a* and biovolume. a: Growth rate of the brine algal incubations with varying pH, based on changes in chl-*a* and biovolume, in November 2012, experiment 1. b: Growth rate of the brine algal incubations (chlorophyll a and biovolume) with constant pH and varying CO_2_ in November 2012, experiment 2. Vertical grey line signifies initial pH and pCO_2_.

**Figure 2:**
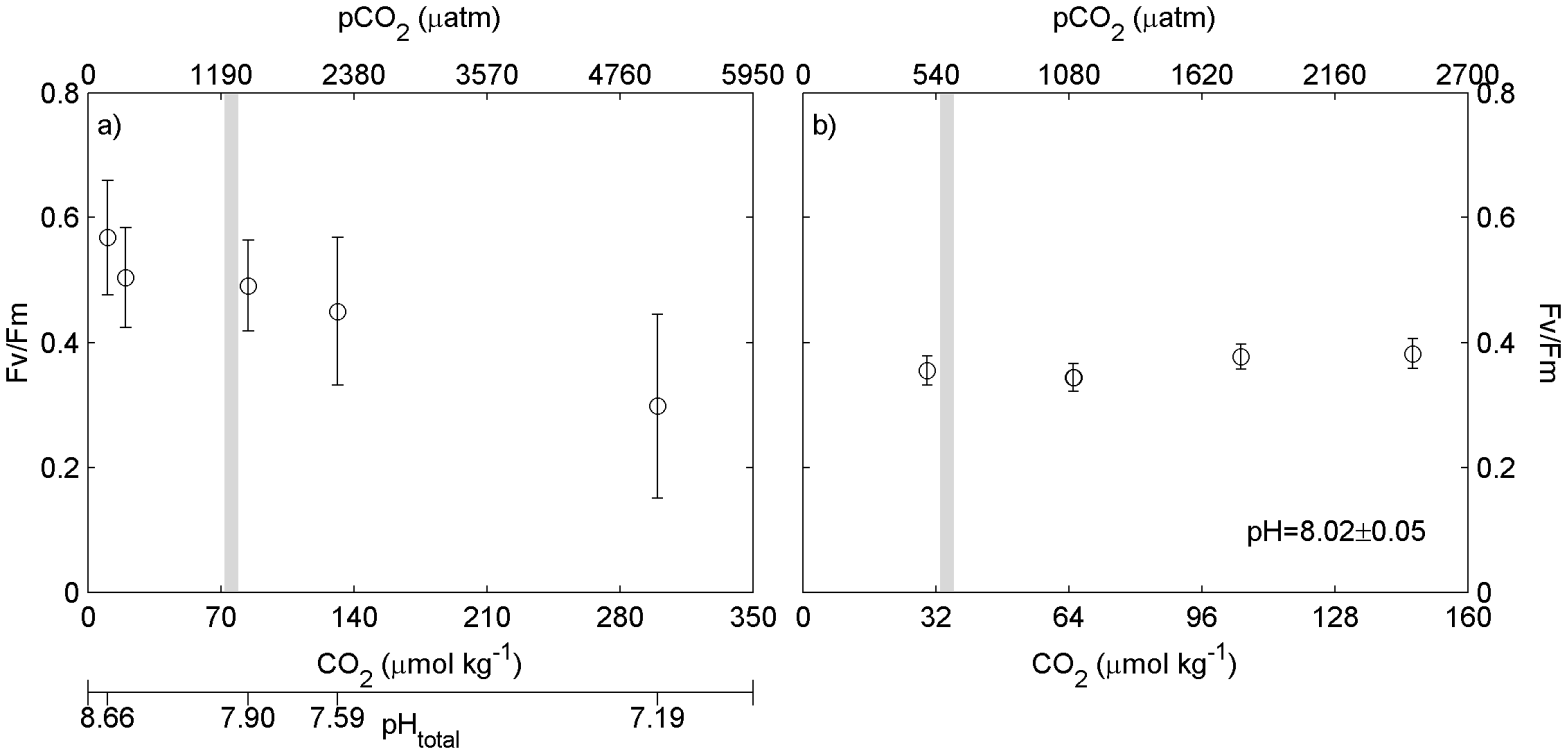
Maximum quantum yield (F_v_/F_m_) of brine algae with varying pH and pCO_2_. a: Maximum quantum yield (F_v_/F_m_) of the brine algal incubations with varying pH in November 2012, experiment 1. b: Maximum quantum yield (F_v_/F_m_) of the brine algal incubations with constant pH and varying CO_2_ in November 2012, experiment 2. Vertical grey line signifies initial pH and pCO_2_.

Initial brine salinity was 65 and the temperature was −3°C. Initial pH and pCO_2_ were 7.84 and 1288 µatm respectively. Manipulated pH and pCO_2_ were between 8.53 to 7.11 and 238 µatm to 6066 µatm respectively (Table 1). Initial chl-*a* concentration in the brine was 0.553±0.058 µg chl-*a* l^−1^ and the initial biovolume of microalgae was 1.36×10^5^ µm^3^/ml. Growth rate estimates from biovolume were consistently higher than estimates from chlorophyll measurements but indicated a similar trend with highest growth rates at pH = 8.37 and lowest at pH = 7.19 (Figure 1a). Initial F_v_/F_m_ was 0.32±0.02. At the end of the incubation values ranged from 0.30±0.15 at pH 7.19 to 0.57±0.09 at pH = 8.66 (Figure 2a) but these differences were not significantly different (p>0.05).

**Table 1:** Carbonate chemistry of CO_2_ manipulation experiments on sea ice brine algae from McMurdo Sound, 2012.

Variable pH experiment. Salinity of 65, temperature of −3°C.							
Initial Brine							
DIC	sd	TA	sd	pH	pCO_2_	CO_3_	CO_2_
3829	2	3938	3	7.84	1288	117	76
**One day after manipulation, DIC and TA values are the means of replicated incubation vials**							
**DIC**	**sd**	**TA**	**sd**	**pH**	**pCO_2_**	**CO_3_**	**CO_2_**
3769	44	4505	7	8.53	238	507	14
3727	5	4293	7	8.40	325	388	19
3766	13	3721	8	7.56	2417	60	142
3618	16	3531	52	7.47	2806	47	165
3518	30	3194	4	7.11	6066	19	357
**End of experiment, DIC and TA values are the means of replicated incubation vials**							
**DIC**	**sd**	**TA**	**sd**	**pH**	**pCO_2_**	**CO_3_**	**CO_2_**
3746	5	4916	23	8.79	117	826	7
3701	13	4232	10	8.37	346	364	20
3478	198	3855	23	8.24	455	257	27
3550	95	3580	5	7.70	1662	78	98
3383	54	3187	27	7.27	4123	27	243
**Constant pH experiment. Salinity of 54, temperature of −2.5°C.**							
**Initial brine (same as first DI treatment)**							
**DIC**	**sd**	**TA**	**sd**	**pH**	**pCO_2_**	**CO_3_**	**CO_2_**
2999	5	3191	3	8.02	587	146	35
**Start experiment**							
**DIC**	**sd**	**TA**	**sd**	**pH**	**pCO_2_**	**CO_3_**	**CO_2_**
2999	5	3191	3	8.02	587	146	35
5785	0	6052	5	7.99	1216	263	72
8635	2	8978	8	7.98	1879	380	111
11396	6	11830	10	7.98	2464	504	146
**End of experiment, DIC and TA values are the means of replicated incubation vials**							
**DIC**	**sd**	**TA**	**sd**	**pH**	**pCO_2_**	**CO_3_**	**CO_2_**
2934	8	3201	2	8.15	423	188	25
5689	26	6031	3	8.07	983	310	58
8554	18	8949	25	8.02	1682	413	100
11300	66	11830	21	7.97	2491	491	148

Units for DIC, TA and CO_3_ is µmol l^−1^. Unit for pCO_2_ is µatm. Standard deviation (sd) is based on five replicates.

### Constant pH (8.02) and Increased CO_2_ Experiment

Initial brine salinity was 54 and brine temperature was −2.5°C. Initial pH and pCO_2_ were 8.02 and 587 µatm respectively. Manipulated pH remained constant at approximately 8.0, manipulated pCO_2_ varied between 587 and 2464 µatm (Table 1). Initial chl-*a* concentration was 5.031 µg chl-*a* l^−1^ and the initial microalgal biovolume was 4.97 10^6^ µm^3^/ml. Estimated growth rates from chlorophyll and biovolume measurements increased with pCO_2_ (Figure 1b). F_v_/F_m_ exhibited no significant difference with respect to CO_2_ concentration (p>0.05, Figure 2b).

## Discussion

OA produces both a drop in pH (and calcium carbonate saturation state) and a rise in CO_2_ concentration and these changes produce quite different results. While a decline in calcium carbonate saturation state primarily impacts calcifiers, an increase in CO_2_ availability potentially impacts photosynthesis in all autotrophs [20], [28]. Bach et al. [28] attempted to isolate the effects of reduced pH and elevated pCO_2_ on the calcifier, *Emiliaia huxleyi*. They found that growth rates were directly related to CO_2_ at low pCO_2_ but at higher CO_2_ levels they were adversely affected by associated decreases in pH. In the experiments described here, the carbonate chemistry was similarly manipulated to identify and then isolate the differing effects of reduced pH and elevated CO_2_ on sea ice brine communities.

The brine algal community was incubated under two different sets of conditions. In the first experiment the alkalinity remained constant and the pCO_2_ was manipulated without controlling the pH. This facilitated pH levels that ranged between 8.66 and 7.19 and pCO_2_ that ranged between 178 and 5095 µatm. Under these conditions growth was maximal at a pH of 8.39 (346 µatm). When the pH was held constant at ∼8.02 and the pCO_2_ manipulated, growth and F_v_/F_m_ continued to increase from pCO_2_ of 505 µatm to 1780 µatm and then remained approximately constant up to 2477 µatm, i.e. growth reached a maximum at four times present atmospheric CO_2_ concentration and remained maximal to at least six times. The results presented here are consistent with those of Bach et al. [29], showing no change in growth rate with increasing CO_2_ at low concentrations but were adversely affected by decreasing pH (<∼7.2) at higher concentrations. These experiments showed that greater falls in pH to approximately 7.2 reduced the growth rate by almost 50%. However, if the pH remained at ∼8.0, increased CO_2_ resulted in a growth rate increase of approximately 20%.

Responses of microalgal taxa in OA experiments have been quite variable. Several recent studies have found that at elevated CO_2_ concentrations (up to 1000 µatm) photosynthesis and growth tends to increase at low irradiances but decrease at higher irradiances [30], [31], [32], [33]. In particular, it has been found that elevated CO_2_ levels cause an onset of photoinhibition at lower irradiances [32]. This response, however, is not universal. While many studies report that diatom growth rates respond positively to moderate increases in pCO_2_ [33], [34], [35], others showed little or no change [36], [37], [38], [39]. With dinoflagellates also, many have shown little response to changes in pCO_2_ that are consistent with end of century scenarios [40].

Some of the taxon-specific differences in response are possibly associated with the possession of an effective carbon concentrating mechanism (CCM). These are widespread in diatoms [41], including sea ice diatoms [42], and have also been reported in some dinoflagellates [43], [44]. However, it is not known whether a CCM is present in *Polarella glacialis*. Dinoflagellates are the only phytoplankton group that possesses form II Rubisco, which is mostly associated with anaerobic phototrophic bacteria, and has a particularly low affinity for CO_2_ [44]. Without a CCM these organisms would experience growth-limiting concentrations of CO_2_ under current oceanic conditions [44] and for brine algae this would be exacerbated in the very high pH conditions often experienced in late spring and early summer. For *P. glacialis* to dominate these highly variable microenvironments, often with very low CO_2_ and O_2_ supersaturation, possession of an effective CCM would be highly likely.

Field studies in the Southern Ocean (Ross Sea) in an area of significant vertical mixing and Fe stress, however, showed a strong relationship between carbon uptake and elevated pCO_2_ [45]. They also found that elevated pCO_2_ caused changes in species composition promoting the growth of large diatoms. Similar community composition shifts were also seen in field experiments in the equatorial Pacific [46].

Plankton communities that are naturally exposed to large variations in pH and CO_2_ concentration, such as brine algae, seem to have a greater tolerance to these changes. This has also been seen, for instance, in some coastal communities of the Baltic Sea [12], [13] and North Sea [14] where natural pCO_2_ varies seasonally between 500 µatm and 2500 µatm. These communities demonstrated a high degree of tolerance and showed only subtle changes to elevated pCO_2_.

Importantly, future marine phytoplankton communities will not only be exposed to decreases in pH but also to coincident increases in abiotic factors such as temperature and light (though increased stratification) and it will be important to consider the cumulative effect of multiple ocean drivers [34], [47], [48]. However sea ice microbial ecosystems are less likely to experience the same changes in abiotic factors as phytoplankton; temperature for instance can never rise beyond 0°C and except for local variations in light caused by snow cover and ice thickness, there are not likely to be significant changes in available light. Including co-varying manipulations of light and/or temperature in the experiments reported here was therefore less relevant than in planktonic systems.

Several of the experimental treatments reported herein experienced a loss of chlorophyll biomass (reported as negative growth rates) during the incubations. The most likely explanation for these decreases is grazing as light and temperature conditions remained unchanged and there is evidence from elsewhere that carbon manipulations do not affect the cellular chlorophyll content [33], [34], [37]. There is, however, evidence that in some ecosystems changes in CO_2_ concentrations affects grazer-prey interactions [9]. Although meiofaunal abundance within sea ice, and within brine channels in particular, is very low [49], [50], there are currently no published measurements of grazing rates. Change in chlorophyll biomass has often been used to measure growth in sea ice ecosystems (e.g. [51]) and there is an unstated assumption that grazing pressure remains constant. This assumption is probably reasonable in this study as incubation periods were relatively short. In this study growth rates were measured both by changes in chl-*a* and also by changes in biovolume. Although the two sets of measurements gave consistent trends, the estimates made by changes in biovolume were consistently higher. While the reason for this is uncertain, one explanation could be due to differential growth rates of different species of algae, e.g. if there were changes in the abundance of large celled diatoms with relatively low chl-*a* content per cell volume, then biovolume in the whole sample would increase at a greater rate than chl-*a*.

The experiments documented here demonstrate that sea ice brine algal communities are tolerant to the changes in pH that are likely to be experienced by the end of the century and will possibly benefit from the associated increases in CO_2_. However, physiological responses and expression of CCM activity are variable between species and even between strains of the same species [44]. Also, the results reported here are based on short incubations and do not account for the ability of species and communities to adapt over longer time periods. It is probable that sea ice brine communities will not be adversely affected by OA.
